# Diagnosis, risk factors, and treatment of pediatric benign pneumoperitoneum: A single‐center retrospective study

**DOI:** 10.1002/pdi3.36

**Published:** 2023-10-30

**Authors:** Yueyue Liu, Quan Kang, Guobin Liu

**Affiliations:** ^1^ General Surgery Department Children's Hospital Affiliated to Xi'an Jiaotong University Xi'an Children's Hospital Xi'an Shaanxi Province China; ^2^ Department of General and Trauma Surgery Children's Hospital of Chongqing Medical University National Clinical Research Center for Child Health and Disorders Ministry of Education Key Laboratory of Child Development and Disorders Chongqing China; ^3^ Molecular Oncology Laboratory The University of Chicago Medical Center Chicago Illinois USA

**Keywords:** benign pneumoperitoneum, children, surgical pneumoperitoneum, visceral perforation

## Abstract

Pneumoperitoneum is one of the common acute abdominal emergencies that leads to surgical exploration in children, but not all pneumoperitoneum require emergency surgery. This study evaluated the diagnostic methods, predisposing risk factors, and therapeutic options in children with benign pneumoperitoneum for accurate diagnosis and reducing the risk of adverse surgical consequences. A total of 63 cases of pneumoperitoneum diagnosed by the radiographs were involved in this study and were divided into the surgical pneumoperitoneum and benign pneumoperitoneum groups, respectively, from 2009 to 2021. The general information, abdominal signs, laboratory examination, therapies and outcomes, total hospitalization time, and comorbidities were analyzed. Logistic regression analysis assessed the risk factors and protective factors of benign pneumoperitoneum. Promising biomarkers underwent receiver operating characteristic curve analysis. Children with surgical pneumoperitoneum were younger than those with nonsurgical pneumoperitoneum in the study. The C‐reactive protein (CRP) level in the surgical pneumoperitoneum group was higher than that in the other group, albumin (ALB) was lower than that in the benign pneumoperitoneum group, and the ratio of ALB to CRP can be used as an effective indicator to predict whether the pneumoperitoneum is benign or whether surgery is needed. Pulmonary injury, pneumonia, and blunt abdominal injury are the major causes in patients with benign pneumoperitoneum. The establishment and management of clinical criteria for the diagnosis of benign pneumoperitoneum in children will be important for the treatment and recovery of children diagnosed with pneumoperitoneum.

## Graphical abstract

The figure shows that not all children with pneumoperitoneum require emergency surgical treatment, and some benign pneumoperitoneum can be clinically improved through non‐surgical treatment. The establishment and management of clinical criteria for the diagnosis of benign pneumoperitoneum in children will be important for the treatment and recovery of pneumoperitoneum.

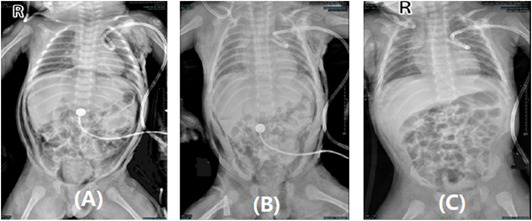

## 1 INTRODUCTION

Pneumoperitoneum is the presence of free gas in the abdominal cavity, and radiological examination of the abdominal cavity is able to confirm it. Approximately 90% of pediatric pneumoperitoneum is due to perforation of the abdominal hollow organs.^1^ These children often have life‐threatening clinical manifestations such as active hemorrhage, severe infection, and even shock. Therefore, once pneumoperitoneum is coupled with the clinical presentation of acute abdomen, surgical intervention should generally be performed. This type of pneumoperitoneum is called surgical pneumoperitoneum; however, approximately 10% of pediatric pneumoperitoneum with stable vital signs, minimal symptoms, and abdominal signs can be treated conservatively. This type of pneumoperitoneum is called nonsurgical, spontaneous, or benign pneumoperitoneum.^2^ However, there may be a situation in which surgeons are unable to determine whether the pneumoperitoneum is surgical or benign, and laparotomy is performed on patients who have no visceral perforation. Patients undergoing surgery have dramatically increased risks of postoperative complications, such as aggravated patient's physical state, elongation of hospital stay and costs as well as other adverse consequences.^2^

As mentioned above, pneumoperitoneum is a radiological diagnosis. It is mainly diagnosed by plain X‐ray film. It usually shows free air under the diaphragm or in a superiorly dependent location on abdominal radiographs.^2^, ^3^ Surgical pneumoperitoneum is mostly accompanied by fever, severe and progressive abdominal pain, and abdominal distension as well as significant peritonitis and even shock.^4^, ^5^ The inflammatory indicators are significantly increased in the laboratory examination reports. Fluid and gas accumulation are visible on abdominal ultrasound.^6^ Abdominocentesis can extract fluid or gas and laparotomy can clearly identify the injured or perforated organ. Children with benign pneumoperitoneum can also present with fever, abdominal pain, abdominal distension, and other discomfort, with or without a peritonitis presentation. Inflammatory indicators can be elevated or remain normal. However, the vital signs of these children are stable. They are generally in a good condition, and their symptoms and abdominal signs are improved after some conservative treatment when the radio‐images show that the air has decreased or has dissolved. Some of them undergo laparotomy; however, the perforated organ is not found.^2^, ^5^

In summary, the clinical manifestations of surgical and benign pneumoperitoneum may be extremely similar at the disease onset. Depending on the clinical manifestations and imaging findings, it may still be difficult to identify them accurately and in a timely manner. This is still a dilemma for clinicians about how to evaluate the patient's condition and decide the mode of treatment.^7^ Some children with benign pneumoperitoneum may have adverse outcomes, such as postoperative complications due to unnecessary surgery. Most of the previous studies of pediatric benign pneumoperitoneum are case reports.^5^, ^8^ Our study retrospectively analyzed the medical records of pneumoperitoneum patients in a single‐center tertiary hospital for children and discussed the diagnosis and treatment of benign pneumoperitoneum in children.

## 2 METHODS

This study was approved by the ethics committee of the Children's Hospital of Chongqing Medical University (ethics approval No 2021‐349).

In this retrospective study, the medical records of pediatric pneumoperitoneum patients admitted to the Children's Hospital of Chongqing Medical University from June 2009 to June 2020 were reviewed.

This study included 63 children diagnosed with pneumoperitoneum by radiographs, including 40 surgical pneumoperitoneum patients who were diagnosed with digestive tract perforation by surgery, and 23 nonsurgical pneumoperitoneum patients. All cases were confirmed by imaging examinations such as thoracic or abdominal X‐ray or abdominal CT scan (free gas shadow was visible in the radio‐images). In this study, 40 children with surgical pneumoperitoneum were diagnosed by imaging at first and then confirmed by surgery; 23 patients with nonsurgical pneumoperitoneum underwent no surgery after imaging and diagnosis of pneumoperitoneum and after they improved by conservative treatment; and gastrointestinal perforation was not identified after surgery. They all had complete recorded medical data. Patients who had abdominal surgery or an invasive operation before the abdominal free gas were included and those with incomplete medical data were excluded.

The above 63 children were divided into two groups: the surgical pneumoperitoneum group and the nonsurgical pneumoperitoneum group. The clinical statistics included general information, abdominal signs, laboratory examination results, therapies and outcomes, and comorbidities. All data of the patients were collected and analyzed by IBM‐SPSS version 24.0. Multivariate analysis was performed by logistic regression analysis, and when *p* < 0.05, the difference was statistically significant. The statistical figures were drawn by GraphPad Prism 8.0.1.

## 3 RESULTS

**FIGURE 1:**
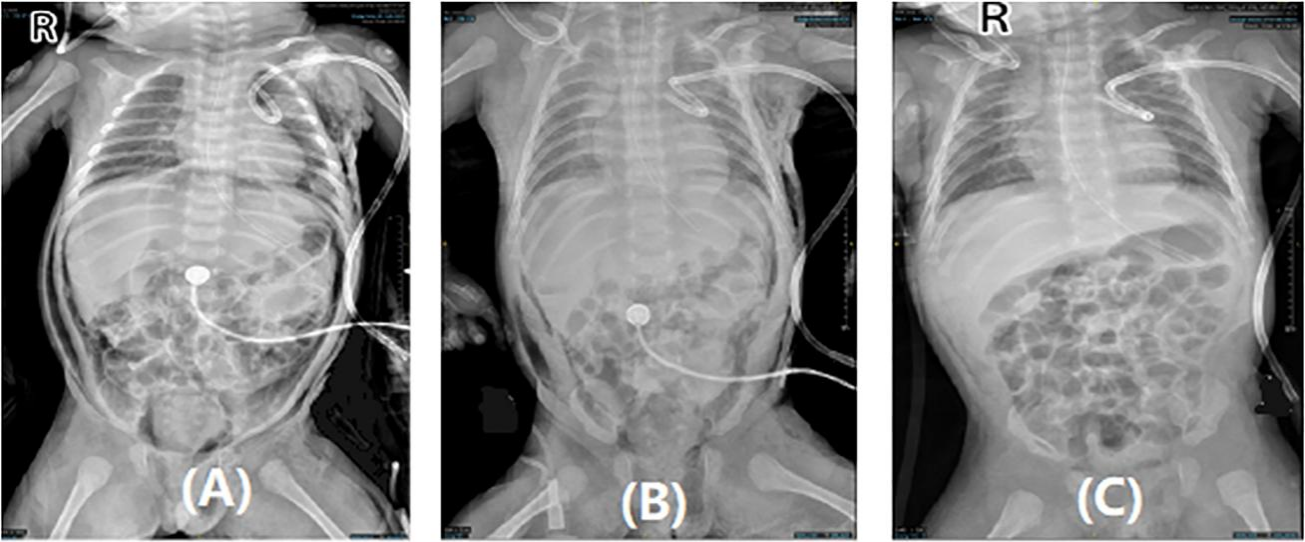
A case in benign pneumoperitoneum group diagnosed by abdominal X‐ray. (A) The radiograph indicating a large amount of pneumoperitoneum. (B) The obvious decrease of abdominal free air after 1 day. (C) Progressive reduction of the air after 5 days.

A total of 63 cases were documented in this study. Thirty‐five male and 28 female patients were included. The diagnosis of pneumoperitoneum was demonstrated in all cases on either plain X‐ray film or CT. These 63 children were divided into two groups: one group comprised 40 patients who were diagnosed with visceral perforation and surgical pneumoperitoneum, and the other group included 23 children who were considered to have benign pneumoperitoneum at discharge. The general information, abdominal signs, detailed laboratory findings, therapies, comorbidities, and other information of all patients who were imaged for pneumoperitoneum are summarized in Table 1. Figure 1 displays the main diagnostic methods for different pneumoperitoneum types is X‐ray, includes three images, which are the abdominal X‐ray plains of a case in the nonsurgical (benign) pneumoperitoneum group during disease.

**TABLE 1:** Analysis of clinical data for patients in surgical pneumoperitoneum group and nonsurgical (benign) pneumoperitoneum group.

	Surgical (*n* = 40)	Benign (*n* = 23)	*p* value
General information			
Gender			
Male:Female	21:19	14:9	0.52
Age (median [min–max]) years old	1 (0–15)	6.08 (0–13.75)	<0.001
<28 days	0.40 (16/40)	0.17 (4/23)	
>28 days	0.6 (24/40)	0.83 (19/23)	
Weight (median [min–max] kg)	8.50 (1.15–35.00)	19.00 (1.50–60.00)	0.001
Days of disease onset (median [min–max]) days	2 (0.04–18)	1 (0.04–60)	0.49
Abdominal sign			
Peritonitis (yes:no)	40:0	7:16	<0.001
Laboratory examination			
WBC (median [min–max] × 10^9^/L)	9.28 (1.26–39.71)	15.23 (3.53–27.94)	0.03
Percentage of neutrophils median (min–max)	0.75 (0.2–0.96)	0.82 (0.43–0.96)	0.04
CRP (mg/L) (mean ± SD, median)	57.90 ± 60.39, 40.00	31.74 ± 57.44, 8.00	0.006
Albumin (g/L)	29.90 ± 7.14	34.70 ± 7.13	0.013
ALB/CRP median (min–max)	0.80 (0.09–6.64)	3.78 (0.12–6.29)	0.001
Therapies and outcomes			
Fasting (yes:no)	40:0	19:4	0.03
Laparotomy (yes:no)	40:0	2:21	<0.001
Fasting duration (median [min–max]) days	6 (2–16)	3 (0–30)	0.001
Time of pneumoperitoneum to disappear (days)	‐	3 (1–13)	
Total hospitalization days (median [min–max]) days	18 (6–64)	14 (2–53)	0.25
Comorbidities			
Pneumothorax	0.03 (1/40)	0.4 (10/23)	<0.001
Mediastinal emphysema	0	0.24 (6/23)	0.002
Blunt injury of abdomen	0.03 (1/40)	0.4 (10/23)	<0.001
Pneumonia	0.18 (7/40)	0.35 (8/23)	0.12
Pulmonary contusion	0	0.35 (8/23)	<0.001
NEC	0.25 (10/40)	0.09 (2/23)	0.21
Dysplasia	0.15 (6/40)	0.04 (1/23)	0.38
Special or severe infection	0.35 (14/40)	0.17 (4/23)	0.14

Abbreviations: ALB, albumin; CRP, C‐reactive protein; NEC, necrotizing enterocolitis; WBC, white blood cell.

The mean age of the children in the surgical pneumoperitoneum group was 1 year old. In this group, 16 newborns were involved with ages ranging from 1 to 23 days. The maximum age was 15 years old. The mean age of the children in the benign pneumoperitoneum group was 6.08 years old, and only four newborns were included, with ages ranging from 1 h to 16 days. The maximum age was 13.75 years old. The difference was statistically significant. Apparently, there was a correlation between children's weights and ages. The median weight of the children in the surgical pneumoperitoneum group was 8.50 kg and ranged from 1.15 to 35.00 kg. The median weight and weight range of children in the benign pneumoperitoneum group were 19.00 and 1.50–60.00 kg, respectively, which were statistically significant. In addition, no statistically significant differences were found in days of disease onset or sex between the two groups in this study.

All 40 children in the surgical pneumoperitoneum group had peritonitis in the course of the disease, a manifestation of abdominal tenderness, abdominal tension, rebound pain, and red and swollen abdominal wall. Among the children in the benign pneumoperitoneum group, 7 children had different types of peritonitis located or generalized peritonitis. There were 16 out of 23 children in the benign pneumoperitoneum group without significant abnormal abdominal signs. There was a statistically significant difference.

Regarding the laboratory examinations of all included patients in our study, the results of leukocyte count (WBC), percentage of neutrophils (N%), C‐reactive protein (CRP), and albumin (ALB) were analyzed. WBC, CRP, and ALB were verified as statistically significant biochemical markers between the two groups. Compared with the patients in the surgical pneumoperitoneum group, the children who were not diagnosed with surgical pneumoperitoneum had higher WBC counts (15.23 [3.53–27.94] × 10^9^/L), ALB levels (34.70 ± 7.13 g/L), and percentages of neutrophils (N%) (0.82 [0.43–0.96]), but lower CRP levels ([mean ± SD, median] 31.74 ± 57.44, 8 mg/L). Based on the above results, CRP and ALB were significantly different between the two groups, and the values of ALB/CRP were compared after. ALB/CRP (0.80 [0.09–6.64]) in the surgical pneumoperitoneum group was lower than that in the benign pneumoperitoneum group (3.78 [0.12–6.29]).

All the children in the surgical pneumoperitoneum group were confirmed to have gastrointestinal perforation and underwent fasting. Four patients in the benign pneumoperitoneum group did not fast because no obvious abdominal signs or clinical symptoms were found. The mean fasting time of the children in the surgical pneumoperitoneum group was longer (6 [2–16] days) than that in the benign pneumoperitoneum group. Only two patients in the benign pneumoperitoneum group underwent laparotomy because of the obvious aggravation of generalized peritonitis; however, no perforated viscus was found during the operation. One patient had concurrent systemic tuberculosis. Intestinal adhesion and incomplete intestinal obstruction were considered postoperative diagnoses. The total hospitalization days were not significantly different between the two groups.

Children diagnosed with pneumoperitoneum had a combination of a variety of complications that may indicate the causes or risk factors for the disease. Apart from gastrointestinal perforation, the children in the surgical pneumoperitoneum group had some comorbidities, such as necrotizing enterocolitis (NEC) (*n* = 10), intestinal obstruction (*n* = 13), special or severe infection (*n* = 14), pneumonia (*n* = 7), abnormal development, or deformity (*n* = 6), and pneumothorax (*n* = 1). Among the children in the benign pneumoperitoneum group, pneumothorax (*n* = 10), mediastinal emphysema (*n* = 6), pneumonia (*n* = 8), pulmonary contusions (*n* = 8), blunt abdominal trauma (*n* = 10), abnormal development or deformity (*n* = 1), special or severe infection (*n* = 4), and suspected NEC (*n* = 2) were observed.

The logistic regression analysis of the children’s age, weight, WBC count, N%, CRP, ALB, and ALB/CRP is shown in Table 2. ALB/CRP was identified as the only statistically significant biochemical marker among the patients in the two groups (*p* = 0.015, OR = 0.504, and CI 0.290–0.876). ALB/CRP may be a protective factor for diagnosing benign pneumoperitoneum.

**TABLE 2:** Logistic analysis of age, weight, WBC, CRP.

	SE	Wald	*p* value	OR	95% CI	
					Lower	Upper
Age	0.224	0.345	0.557	0.877	0.565	1.360
Weight	0.076	0.184	0.668	0.968	0.834	1.123
WBC	0.050	0.000	0.987	0.999	0.905	1.103
N%	2.659	0.045	0.831	0.567	0.003	104.10
CRP	0.007	0.240	0.624	0.996	0.982	1.011
ALB	0.053	0.065	0.799	0.987	0.890	1.094
ALB/CRP	0.282	5.911	0.015	0.504	0.290	0.876

Abbreviations: ALB, albumin; CRP, C‐reactive protein; N%, percentage of neutrophils; WBC, white blood cell.

**FIGURE 2:**
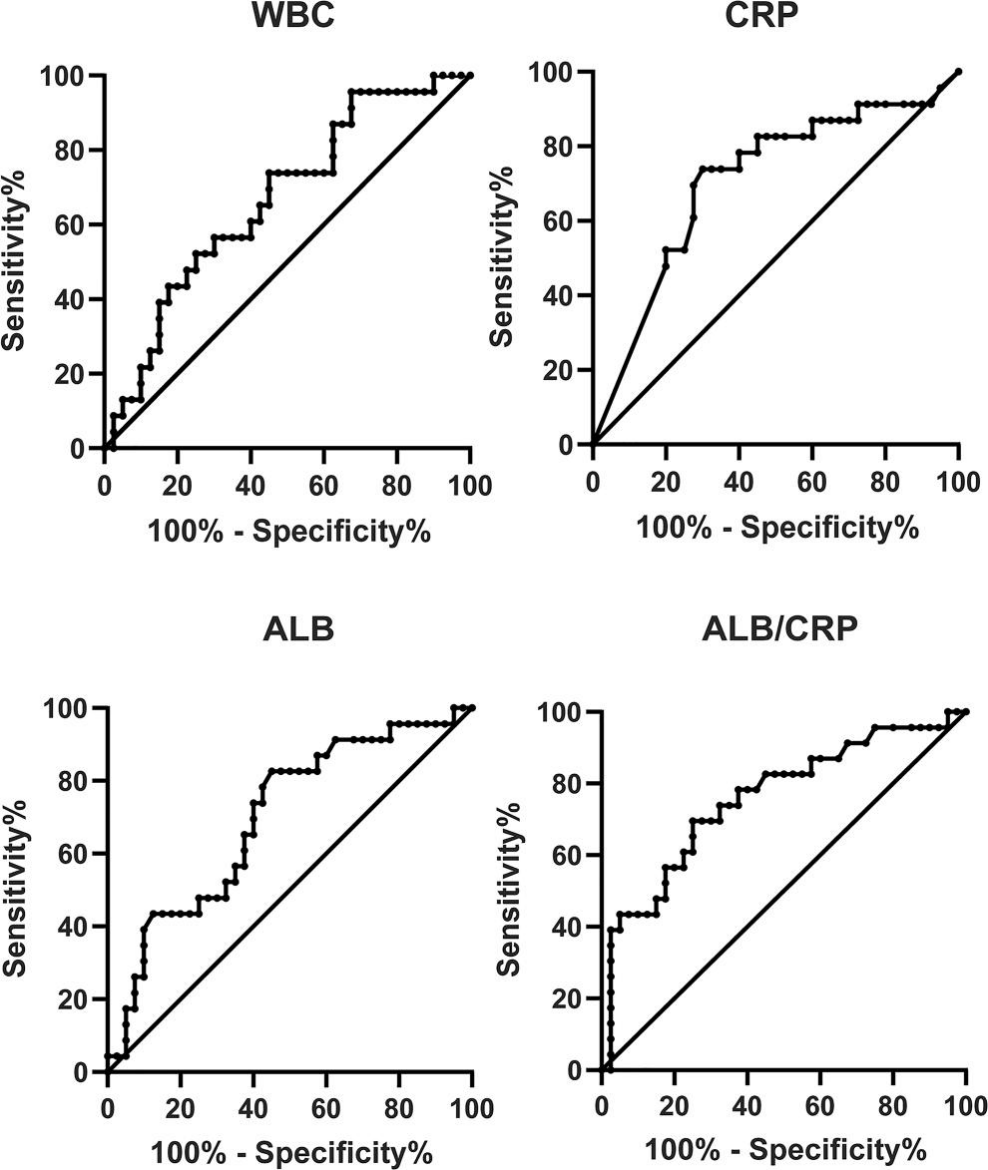
ROC curves for WBC, CRP, ALB, and ALB/CRP. WBC, CRP, ALB, and ALB/CRP are verified as statistically significant biochemical markers between surgical pneumoperitoneum group and nonsurgical (benign) pneumoperitoneum group. The ALB/CRP level is a better predictor of distinguishing the pneumoperitoneum type. ALB, albumin; CRP, C‐reactive protein; WBC, white blood cell.

According to the receiver operating characteristic (ROC) curve analysis of WBC, CRP, ALB, and ALB/CRP (Table 3), the area under the ALB/CRP curve, as shown in Figure 2, was 0.76 and *p* < 0.01 with a sensitivity of 70% and specificity of 75%. The optimum cutoff was 2.65, suggesting that ALB/CRP was a better predictor of distinguishing the pneumoperitoneum type, which means that the larger the value of ALB/CRP is, the more likely the pneumoperitoneum is to be nonsurgical or benign. Additionally, the areas under ROC curve (AUC) of WBC was 0.67 with a sensitivity of 65% and specificity of 57%, and the optimum cutoff was 10.5. The AUC of ALB was 0.70 with a sensitivity of 83% and specificity of 55%, and the optimum cutoff was 29.6. The AUC of CRP was 0.71 with a sensitivity of 70% and specificity of 74%, and the optimum cutoff was 16.5. However, this result still needs further validation by more studies with many cases.

**TABLE 3:** ROC analysis of WBC, ALB, CRP, and ALB/CRP.

	AUC	SE	Optimum cutoff	*p* value	Sensitivity	Specificity	95% CI	
							Lower	Upper
WBC	0.666	0.06969	>10.5	0.0289	65%	57%	0.5297	0.8029
ALB	0.697	0.06837	>29.6	0.0096	83%	55%	0.5633	0.8313
CRP	0.707	0.07021	<16.5	0.0067	70%	74%	0.5689	0.8441
ALB/CRP	0.758	0.06515	>2.65	0.0007	70%	75%	0.6305	0.8858

Abbreviations: ALB, albumin; AUC, areas under ROC curve; CRP, C‐reactive protein; WBC, white blood cell.

As we have found in this study, different etiologies exist in children with benign pneumoperitoneum. The children with benign pneumoperitoneum were divided into 5 different stages. As shown in Table 4, in our study, pulmonary injury, pneumonia, and blunt abdominal injury were the major causes in patients with benign pneumoperitoneum.

**TABLE 4:** Etiologies of patients with benign pneumoperitoneum at different ages.

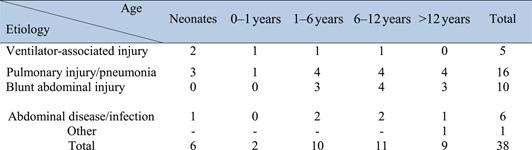

**FIGURE 3:**
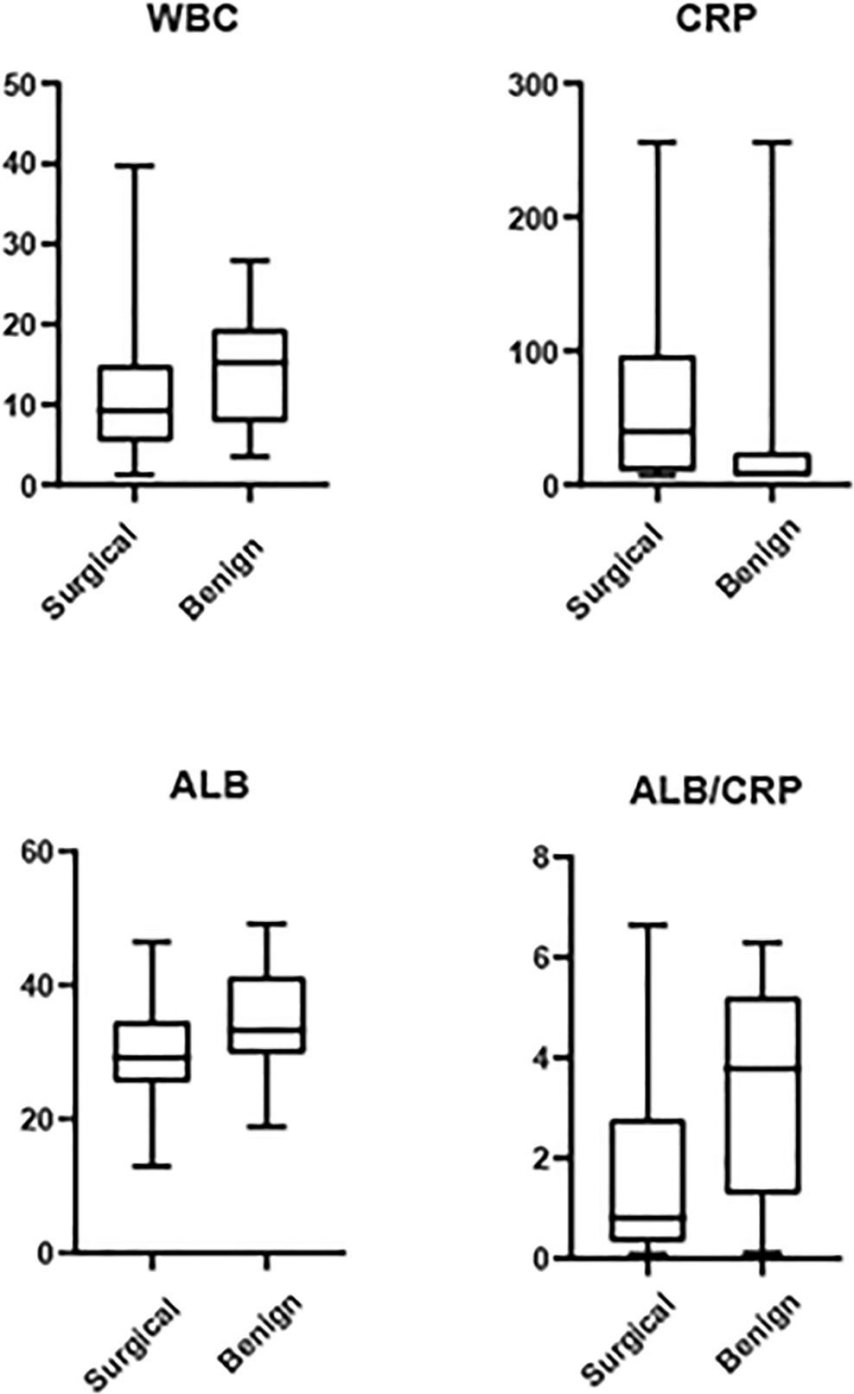
Box and violin graphs for WBC, CRP, ALB and ALB/CRP. The median, IQR, and range for CRP, ALB, WBC, and ALB/CRP values for both groups combined. ALB, albumin; CRP, C‐reactive protein; IQR, interquartile range; WBC, white blood cell.

Figure 3 shows the median, interquartile range, and range for CRP, ALB, WBC, and ALB/CRP values for both groups combined.

## 4 DISCUSSION

Pediatric pneumoperitoneum is diagnosed by radiological examination in which free gas is found in the abdominal cavity. More than 90% of pediatric pneumoperitoneum is caused by perforation of the digestive tract, and it is generally considered a surgical emergency. However, nearly 10% of pediatric pneumoperitoneum clearly diagnosed by radiographs can be treated conservatively.^5^, ^9^, ^10^ Children with only mild relevant clinical manifestations and abdominal signs are considered to be in good condition. After conservative treatment, close observation, and imaging follow‐up, the pneumoperitoneum will dissolve. This kind of pneumoperitoneum is considered benign pneumoperitoneum or nonsurgical pneumoperitoneum.

The etiology for the formation of most benign pneumoperitoneum in children remains unclear.^11^ According to the relevant literature, it is currently believed that the formation of benign pneumoperitoneum in children may be due to the following reasons^2^, ^5^, ^12^: (1) a thoracic injury or a disease such as pneumonia, pneumothorax, mediastinal emphysema, etc.^13^; (2) a ventral disease or an injury such as blunt abdominal injury, intestinal obstruction, inflammation, etc.; (3) obstetric and gynecological conditions such as reproductive tract disease or injury; (4) iatrogenic conditions such as mechanical ventilation, peritoneal dialysis, cardiopulmonary resuscitation, etc.^14^; and (5) other reasons. In our study, 6 patients were diagnosed with pneumoperitoneum after mechanical ventilation; 10 children were diagnosed after abdominal blunt injury; 3 children were diagnosed with pneumonia at the same time; and 3 of the 23 patients were diagnosed with intestinal obstruction due to the clinical characteristics and radio‐images. Only one patient was found to have free abdominal gas after long‐term peritoneal dialysis.^15^ The clinical diagnoses of both pneumothorax and pneumoperitoneum after mechanical ventilation are mostly considered as air leakage syndrome. It easily occurs in newborns.

The genesis of benign pneumoperitoneum in children of different ages may be specific. For example, in this study, the benign pneumoperitoneum of newborns was mostly thoracic in origin (mechanical ventilation, lung lesions); however, pneumoperitoneum occurring in the neonatal period needs to be diagnosed vigilantly. The perforation of intestinal necrosis caused by NEC needs to be excluded first. It is prudent to identify whether the neonatal pneumoperitoneum is benign or a surgical situation.

According to AI‐lawama M et al.'s case report on neonatal pneumoperitoneum, the differential basis for benign pneumoperitoneum and surgical pneumoperitoneum in newborns is summarized (i.e., weight >1500 g, gestational age >32 weeks, time for finding pneumoperitoneum is <5 days, no developmental abnormalities of the gastrointestinal tract, no other abnormalities except free gas in the abdominal cavity, and possible pneumothorax), and benign pneumoperitoneum are often considered higher in neonates with no progressive exacerbation.^16^ Benign pneumoperitoneum in children older than 1 year old is mostly caused by lung lesions or abdominal blunt trauma. Usually, children with abdominal closed injuries may have a pulmonary injury and pneumothorax, and conservative treatment should be considered at first if there is no clear evidence of perforation of organs.

According to previous case reports of pediatric benign pneumoperitoneum, most of these children were generally in good condition; abdominal distension can be the primary or the main symptom; the radiographs show the pneumoperitoneum sign; no effusion occurred from peritoneocentesis (or a small amount of clear fluid was obtained); there was reduction or disappearance of the abdominal free gas after conservative treatment; and surgical pneumoperitoneum should be eliminated as much as possible.^5^ In this study, it was clear that most children with surgical pneumoperitoneum had fever, abdominal pain, and other discomfort. The infectious symptoms were obvious and could be recurrent or even progressively aggravated. All of the patients were presented with peritonitis. However, among 23 children with pneumoperitoneum without perforation of the digestive tract, fever, abdominal pain, abdominal distension, vomiting, shortness of breath, and other discomfort were also the main clinical manifestations, and the symptoms were milder than the children in the perforated group. In addition, the 23 patients showed a shorter duration of the disease course, no progressively aggravated or recurrent symptoms, and the pneumoperitoneum improved or disappeared after conservative treatment. Meanwhile, the corresponding symptoms and signs also improved. Therefore, these cases were regarded as benign pneumoperitoneum. However, seven of these children also showed peritoneal irritation signs; thus, peritonitis can be considered. Two of those who had generalized peritonitis underwent exploratory laparotomies, and no specific perforation of visceral organs was found during the surgery. Five patients were considered to have localized peritonitis. This study indicates that peritonitis is probably relevant for evaluating whether the pneumoperitoneum is surgical or benign. However, children may not cooperate with physical examination sometimes, and in this circumstance, clinicians may not be able to assess the abdominal signs accurately. Therefore, for the identification of pediatric pneumoperitoneum, the evidence is not sufficient when only the abdominal sign is considered. It is necessary to assist the diagnosis with the patient's medical history, laboratory examination, and imaging basis.

In this study, the results of CRP and ALB revealed significant differences in the identification of pediatric pneumoperitoneum. CRP is an acute‐phase response protein synthesized by the liver and is involved in the process of the systemic inflammatory immune response. The higher the elevated CRP level in the short term, the heavier the acute systemic inflammatory response may be. The significant increase in preoperative CRP levels in the perforated group suggests that CRP may be related to the identification of different pneumoperitoneum. In addition, the remarkable reduction in ALB hints that it is more likely to be surgical pneumoperitoneum. ALB is related to the organism's nutritional status and inflammatory infection status. Studies have shown that serum ALB enhances catabolism when the body is deteriorating. The capillaries are impaired due to inflammation, which results in increased permeability, and ALB enters the tissue gap and has a shortened half‐life. Previous meta‐analyses in severe patients suggest that reduced ALB levels are associated with all‐cause mortality in patients.^17^, ^18^, ^19^ Therefore, when the patient is diagnosed with surgical pneumoperitoneum and is in a serious condition, due to abdominal cavity infection and even systemic infection poisoning caused by perforated visceral organs, the ALB level may be reduced accordingly. Apparently, our study suggests that the ALB level may have certain relevance to the diagnosis and identification of pediatric pneumoperitoneum. Thus, the ratio of ALB to CRP may be considered a predictive factor for identifying pneumoperitoneum in children. Our results indicate that the higher the ratio of the two, the more likely the pneumoperitoneum will be benign pneumoperitoneum. An area of 0.76 under the ROC curve indicated the good predictive ability of the ALB/CRP predictive model.

The diagnosis of pneumoperitoneum was clearly defined by imaging examination. Depending on the condition of the child and the related medical history, clinicians can choose different imaging examinations. The primary examination is abdominal or chest plain X‐ray film, which can clarify 55%–85% of pneumoperitoneum cases. The imaging features of thoracic or abdominal standing plain films are mostly manifested as subdiaphragmatic free gas. The abdominal supine and lateral images may show a Rigler sign, and a cross‐table lateral radiograph of the abdomen can show a triangular sign.^20^, ^21^ The diagnostic accuracy of abdominal plain X‐ray film is high and it is more efficient. The children may receive a small amount of radiation, and the plain X‐ray film is more affordable. In this study, abdominal CT examination was mostly used in children with abdominal trauma. CT has higher sensitivity for pneumoperitoneum diagnosis, and it can clarify the injured location of the abdominal organs.^2^, ^14^, ^20^ On the other hand, because of the higher radiation volume as well as the expensive price, it is not suitable for children to undergo repeated examinations in the short term.^2^ At present, ultrasound can also be used to diagnose pneumoperitoneum. Whether there is an abdominal fluid accumulation and the viscera situation can be known simultaneously on ultrasound presentation, which is helpful to identify whether a perforation exists.^6^ In this study, 15 out of 23 patients in the imperforated group underwent abdominal ultrasound, 4 patients had no obvious abnormal sonogram, 6 patients had a small amount of abdominal fluid, 2 patients had low to moderate amount of abdominal fluid, 1 patient had a sonogram indicating intestinal effusion, 2 patients had abdominal wall subcutaneous pneumatosis, and 1 patient had imaging that suggested intestinal flatulence. However, the diagnostic accuracy and efficiency sometimes depend on the ultrasound physician. For younger children in the nonsedative state, an ultrasound examination cannot be performed well, and the credibility of the results is reduced. Therefore, abdominal X‐ray plain imaging is still recommended as the first choice. After the imaging review of 21 children without surgery in this study, the mean time for significant reduction or disappearance of intra‐abdominal gas was 3 days, with a minimum of 1 day and a maximum of 13 days. Of course, this is closely related to the interval of imaging follow‐up.

Case reports have shown that the possibility of the occurrence of benign pneumoperitoneum may be higher in newborns.^2^, ^22^, ^23^ Only five newborns were included in this study, possibly because the number of cases in this group was small. In addition, if newborns are diagnosed with pneumoperitoneum, NEC is the first disease that is considered in most cases. The imperfect neonatal immune system, poor development of abdominal wall muscles, low physical specificity, and sensitivity of laboratory indicators remain great challenges for clinicians to diagnose and identify. Large sample studies and prospective studies are necessary.

Conservative treatment can be considered for children with good general conditions, mild symptoms, and abdominal signs. Considering the laboratory examination results at admission (the inflammatory indicators such as WBC and CRP are not rising substantially, without ALB obviously reducing), these children with pneumoperitoneum can be treated with close attention regarding jejunitis, fluid infusion, nutritional support, antibiotics, and abdominal cavity puncture when necessary. Regular follow‐up imaging examinations were performed to clarify the abdominal status.^24^ A comprehensive evaluation of the patient's clinical symptoms, abdominal signs, lab examination results, imaging examination, etc., should be used to decide whether laparotomy is needed. If the condition improves and the free gas in the abdominal cavity disappears after conservative treatment, the diagnosis of benign pneumoperitoneum is verified. We attempted to determine the etiology to prevent recurrence as much as possible. If during the conservative treatment process, the patient's symptoms and abdominal signs deteriorated, if the air in the abdomen continues to be seen, and if there is no improvement even with a trend of increase, elevated inflammatory indicators, and reduced ALB level, surgical exploration could be actively performed. Patients at a young age, especially neonates, should be given more attention due to the nontypical presentations and the faster variation of disease. Therefore, the diagnosis of benign pneumoperitoneum in children must be as clear as possible after excluding the possibility of visceral perforation. In other words, pediatric benign pneumoperitoneum is an exclusive diagnosis.

### 4.1 Limitations

The number of cases considered with benign pneumoperitoneum in this study is small, and the results of the study require further validation.

There were fewer neonatal pneumoperitoneum cases included in this study and no further intensive study was available.

### 4.2 Suggestions for ongoing research

A multicenter study involving the prospective analysis of clinical factors mentioned in our study in children with pneumoperitoneum was conducted to identify benign pneumoperitoneum.

Looking further at biochemical markers such as procalcitonin and IL‐6.

The number of cases should be increased to further verify the results of this study.

Stratified studies can be performed on children with benign pneumoperitoneum of different ages.

## 5 CONCLUSION

For children with stable vital signs, minimal clinical manifestations and abdominal signs without progressive deterioration, benign pneumoperitoneum should be considered. In addition to the medical history and imaging findings, ALB/CRP can be used as an effective indicator to predict whether surgery should be performed. For children with a history of mechanical ventilation, lung disease or injury, and closed abdominal injury, the possibility of benign pneumoperitoneum should be accounted for, but the possibility of surgical pneumoperitoneum should still be excluded as much as possible. If the likelihood of benign pneumoperitoneum is greater than that of surgical pneumoperitoneum, conservative treatment is preferred.

## AUTHOR CONTRIBUTIONS

Yueyue Liu designed the study and played an important role in data collection and statistical analysis, also responsible for writing. Quan Kang conceived of the study and was responsible for part of the data collection and statistical work. Guobin Liu was responsible for review and revision. All authors reviewed and revised the manuscript and approved the final version.

## CONFLICT OF INTEREST STATEMENT

The authors declare that the research was conducted in the absence of any commercial or financial relationships that could be construed as a potential conflict of interest.

## ETHICS STATEMENT

This study was approved by the ethics committee of the Children's Hospital of Chongqing Medical University (ethics approval No 2021‐349).

## Data Availability

The data that support the findings of this study are available from the corresponding author upon reasonable request.
